# Shortening Fermentation Period and Quality Improvement of Fermented Fish, *Chouguiyu*, by Co-inoculation of *Lactococcus lactis* M10 and *Weissella cibaria* M3

**DOI:** 10.3389/fmicb.2018.03003

**Published:** 2018-12-17

**Authors:** Ruiqi Bao, Sasa Liu, Chaofan Ji, Huipeng Liang, Song Yang, Xiaoming Yan, Yingqin Zhou, Xinping Lin, Beiwei Zhu

**Affiliations:** ^1^National Engineering Research Center of Seafood, Dalian Polytechnic University, Dalian, China; ^2^Institute of Agro-products Processing, Anhui Academy of Agricultural Science, Hefei, China

**Keywords:** fermented fish, starter cultures, flavor, E-nose, GC-MS, MALDI TOF-MS, quality

## Abstract

*Chouguiyu*, a Chinese traditional fermented fish, is famous for its uniquely strong odor and desirable taste. However, traditional spontaneous fermentation often resulted in contamination and unstable quality of products. In this study, individual or conjunctive inoculation of two indigenous lactic acid bacteria (LAB), *Lactococcus lactis* M10 and *Weissella cibaria* M3, was tested for their effect on improving *Chouguiyu*’s quality. It was shown that inoculation would not affect the system’s pH, while increased the total bacteria count and lactic acid bacteria amounts. Matrix-assisted laser desorption/ionization time-of-flight mass (MALDI-TOF) analysis results revealed that *Lactoc. lactis* M10 and *W. cibaria* M3 could quickly occupy a dominant position in the ecosystem, and *Lactoc. lactis* M10 played an important role in the control of spoilage bacteria. Volatile basic nitrogen (TVB-N), thiobarbituric acid reactive substances (TBARS), and biogenic amines results also showed that *Lactoc. lactis* M10 had a positive effect on improving the product’s quality. Co-inoculation of *Lactoc. lactis* M10 and *W. cibaria* M3 could promote the formation of flavor according to the E-nose and gas chromatography-mass spectrometer (GC-MS) analyses, especially for the aroma-active and key volatile compounds. PCA plots of E-nose and hierarchical clustering analysis of GC-MS profiles revealed that the co-inoculation sample at the fifth day (LW5) was the most similar to the natural fermentation sample at the seventh day (C7). The overall acceptance of LW5 was also the closest to that of C7 in sensory evaluation. In conclusion, mixed starter culture was shown to have a good effect on improving product quality and enhancing flavor with fermentation time shortened by 29%.

## Introduction

*Siniperca chuatsi* is a widely cultivated fish in China, and is of great commercial value (Yang et al., 2016). However, due to the rapid deterioration of fresh *Si. chuatsi* meat, fermentation process was developed and resulted in a well-known fish product called *Chouguiyu*, also known as stinky mandarin fish. Similar to a traditional Chinese soybean food—stinky tofu, this special fermented fish has a uniquely strong, smelly odor but desirable taste, and is widely welcome by the majority of consumers in China. Traditionally, the fresh fish *Si. chuatsi* was killed and cured with spices and salts, and then stored at 12 ± 4°C for 7–12 days in jars with heavy stones on the top layer of the fish, and the jars were sealed with covers (Yang et al., 2017).

It was known that a spontaneous fermentation process was characterized by a complex community of microorganisms, which was important for the degradation of substrates, such as protein and carbohydrate, and gradual formation of fermented flavors (Thierry et al., 2016). In the spontaneous fermentation process, it took a long time for the system to form the dominant microbial community. This has become the bottleneck for industrial production of most traditional fermented foods. Besides, the natural microbial community was easily influenced and contaminated by the environmental conditions, such as variable temperature, spoilage, and pathogen bacteria. Manufacture factors, such as moisture or temperature (Yang et al., 2017), had been investigated for their effect on the *Chouguiyu*’s quality, while it was still difficult to produce fermented fish with stable quality. The diversity of lactic acid bacteria involved in *Chouguiyu* fermentation had been investigated by Dai et al. (2013), and *Lactobacillus sakei* was found as the dominant species during the fermentation process. However, little research has been done on selecting starter culture to improve the *Chouguiyu*’s quality.

Inoculation of starter culture is a common strategy to improve the quality of fermented foods (de Almeida et al., 2018; Xu et al., 2018). Compared with spontaneous fermentation, inoculation of starter culture into fermented foods possesses more advantages, such as shortening fermentation time, delaying spoilage, promotion of flavor, and improving product’s quality. Lactic acid bacteria (LAB), known as the most popular starter culture, have been widely applied in various fermentation process of foods, such as meat (Kargozari et al., 2014), fish (Zeng et al., 2013; Gao et al., 2018), and cheese (Ruggirello et al., 2018) manufacture. It had been reported that indigenous LAB originated from the fermented fish *Suanyu* were able to quickly adapt to the ecology, and inhibit the growth of spoilage bacteria and pathogens as well as the increase of thiobarbituric acid reactive substances (TBARS) and volatile basic nitrogen (TVB-N) (Zeng et al., 2013). Gao et al. (2018) found that the inoculation of *Lactob. plantarum* could accelerate the formation of the flavors, especially the esterification and alcoholysis biosynthesis pathway in fermented fish. Biogenic amines accumulation could also be reduced by *Lactob. plantarum* during the fermentation of silver carp sausage (Zhang et al., 2013). According to previous study on *Chouguiyu* (Ji et al., 2017), LAB were discovered in fermented fish and were found to have impact on enhancing carbon metabolic pathway, which might have a function in the flavor formation.

In this study, the microbiological, physicochemical, volatile, and sensory properties of *Chouguiyu* with single starter culture (*Lactococcus lactis* M10, *Weissella cibaria* M3), mixed starter culture (*Lactoc. lactis* M10 and *W. cibaria* M3), and without starter culture (the control) were evaluated. The purpose of this study was to enhance the quality of product and shorten fermentation time by inoculation of starter cultures.

## Materials and Methods

### Starter Cultures

In the beginning of doing this experiment, nine isolated LAB from *Chouguiyu* were used to inoculate the fish and the sensory evaluation was performed. Two bacteria, *Lactoc. lactis* M10 (CGMCC 16612) and *W. cibaria* M3 (CGMCC 16611), showed higher sensory scores than the other strains (unpublished results). These two strains were identified by 16S rDNA analysis with primers of 27F (5′-AGAGTTTGATCCTGGCTCAG-3′) and 1492R (5′-GGTTACCTTGTTACGACTT-3′). *Lactoc. lactis* M10 and *W. cibaria* M3 were cultured in MRS broth (Qingdao Hope Bio-Technology Co., Ltd) at 37°C for 18 h. Cell pellets were harvested by centrifuging at 10,000 × *g* for 2 min at 4°C. Subsequently, they were washed twice with saline water (0.9% NaCl) and re-suspended in 15-ml sterile water before use.

### Chouguiyu Sample Preparation

Fresh *Si. chuatsi* (average weight 600 ± 100 g) were purchased from Changxing market in Dalian, China. Fish were stored in ice within 24 h after being caught from the fish grounds. Then, the fish were gutted and prepared according to the traditional techniques by soaking the fish in salt (6% w/v) water with fennel (0.02% w/w), cumin (0.04% w/w), anise (0.06% w/w), Chinese prickly ash (0.03% w/w), chilli powder (0.002% w/w), ginger (0.6% w/w), and shallot (1.0% w/w). Then, the fish and salt water were divided into four batches and inoculated with different starter cultures, including L (*Lactoc. lactis* M10), W (*W. cibaria* M3), LW (*Lactoc. lactis* M10 and *W. cibaria* M3, 1:1), and a batch without any starter (C) as the control. Each starter culture (7 log cfu ml^−1^) was inoculated into the curing liquid and thoroughly mixed. The fish were covered with heavy bags (average weight of 7 kg for each fish). Fish containers were covered and stored at 12 ± 1°C for 5–7 days. Samples were collected on days 1, 3, and 5 for microbiological, physicochemical, and volatile analyses. Sensory evaluation was carried out at the end of the process.

### Microbiological Analysis

Liquid sample of volume 1 mL was aseptically transferred to a sterile test tube with 9 ml of sterile normal saline (0.9% NaCl). Serial 10-fold dilutions were performed with the same normal saline, and 0.2 ml of each dilution was inoculated in different growth media for microbial counting. LAB on deMan, Rogosa, and Sharpe (MRS, QingDao Hope Bio-technology Co., Ltd) agar was incubated at 37°C for 36–48 h and plate count agar (QingDao Hope Bio-technology Co., Ltd) for total bacteria count was incubated at 37°C for 36–48 h (Essid and Hassouna, 2013). The results were shown as colony-forming units per milliliter (cfu/ml). At the end of cultivation, the fresh colony from MRS and plate count agar plates was used to identify the microbial diversity with matrix-assisted laser desorption/ionization time-of-flight mass spectrometry (MALDI TOF-MS) (Bruker, Autoflex, Germany) according to the previous reports (da Cruz Pedrozo Miguel et al., 2017; El Hamzaoui et al., 2018). Briefly, a colony was scraped from the plate and placed on a steel target plate. Then, 1 μl of 70% formic acid was directly covered onto the colony. After air drying, 1 μl of matrix solution (50 μl acetonitrile, 47.5 μl water, 2.5 μl trifluoroacetic acid, and 1 mg α-cyano-4-hydroxycinnamic acid) was added to each spot and air dried. This colony was analyzed by MALDI TOF-MS. Assays were carried out on Microflex LT bench-top mass spectrometer with FlexControl 3.0 software.

### Determination of pH, TVB-N, and TBARS

The pH value of fermentation liquid was measured by a pH meter (MettlerToledo Instruments Co., Ltd., Shanghai, China). TVB-N content was determined by Cobb’s method (Cobb et al., 1973). The TVB-N concentration was expressed as mg/100 g fish. TBARS assay was carried out according to John’s report (John et al., 2005) and TBARS concentration was expressed as mg/kg.

### Biogenic Amine Determination

Biogenic amine contents of *Chouguiyu* samples at the end of fermentation were analyzed according to a previous report (Zhang et al., 2013). Biogenic amines were extracted with 0.4 M perchloric acid and then the extractions were derivatived with dansyl chloride according to Mah’s report (Mah et al., 2002). Hypersil ODS C18, 5 mm, 4.6 × 200 mm column (Thermo, Bellefonte, PA, USA) was used to separate the derivatives with temperature of 35°C and flow rate of 1 ml/min. Mobile phase A (0.01 M ammonium acetate) and mobile phase B (90% acetonitrile) were used in a gradient elution program. The program was started at 45% A and 55% B, and then raised to 95% B within 25 min and held for 10 min. Biogenic amines were detected at 254 nm and expressed in mg/kg.

### Volatile Compounds Analysis by E-Nose and GC-MS

The volatile compounds were analyzed by the E-nose (PEN3, Airsense, Germany) and their concentrations were determined by solid-phase gas chromatography-mass spectrometer (GC-MS) equipped with micro-extraction (HS-SPME) according to Li’s study (Li et al., 2013). For electronic nose detection, the samples were taken in 40-ml vials and heated at 60°C for 30 min. The measurement phase was set for 70 s until the sensors reached stable values. The performance characteristics for the 10 different metal oxide sensors are listed in Table S2. For SPME, a fiber with 50/30 μm divinylbenzene/carboxen/polydimethylsiloxane (DVB/CAR/PDMS; Supelco Inc., Bellefonte, AL, USA) was used to extract volatile compounds by stirring at 60°C for 70 min. The adsorbent volatiles were thermally desorbed at 260°C for 5 min. Volatiles were separated by a capillary column HP-5MS (30 m × 250 μm × 0.25 μm, Agilent Technologies Inc., California, USA). Helium was used as a carrier and its flow rate was 1.5 ml/min. The initial temperature was 30°C for 5 min; thereafter, the column temperature was raised at a rate of 3°C/min to 50°C (held 3 min), then 5°C/min to 150°C, and finally 20°C/min to 250°C (held 5 min). Retention indices (RIs) were calculated by determining retention time of a mixture of n-alkanes (C_5_–C_20_) under same GC conditions with samples. Volatiles were semi-quantified by 2,4,6-trimethylpyridine as an internal standard and identified based on comparison of mass spectral data with GC-MS library (NIST11, Agilent Technologies, Inc., Santa Clara, CA).

### Sensory Analysis

Samples were evaluated by sensory evaluation to assess the differences between control group (fermented for 7 days) and samples inoculated with starter cultures (fermented for 5 days) by a taste panel composed of 10-member food professional students. A preparatory session was held prior to the evaluation to ensure that each panelist could thoroughly discuss and clarify *Chouguiyu*’s attributes. A 10-point hedonic scale, in which a score of 1 equals dislike extremely, 5 equals neither like nor dislike, and 10 equals like extremely, was used for evaluation. The evaluations were performed in individual booths. The panelists were asked to evaluate the appearance, aroma, taste, tactile texture, and overall acceptance. The results of sensory scores were calculated as the mean value of panelists’ scores.

### Statistical Analysis

Statistical analysis was carried out using SPSS (version 22.0, IBM, USA). The results were expressed as mean ± SD and analysis of variance (ANOVA) was used. When *p* < 0.05, the variation was considered to change the dependent variables significantly. Analyses of data collected from the E-nose were performed by WinMuster (Airsense, Germany). Principal component analysis (PCA) was used to investigate the sensor data between the control and samples inoculated with starter cultures using Canoco for Windows 4.5 software (Wageningen UR, Netherlands). R (version 3.4.4) was used to design the thermography and cluster analysis.

## Results and Discussion

### Changes of pH, Total Bacteria, and Lactic Acid Bacteria During the Fermentation

The initial pH ranged from 6.81 to 6.97 in the control and inoculated samples (Table 1). During the fermentation process, pH values decreased slightly and fluctuated between 6.47 and 6.58 in all samples. It was also observed in traditional Korean fish sauce saeu-jeot (Lee et al., 2014), in which pH gradually dropped to 6.9–7.0 at the first 10 days. Zeng et al. (2016) also reported the pH value was about 6.0 at the first 10 days’ fermentation in *Suanyu*, a traditional fermented fish in China, with LAB as starter culture. Fermented foods usually have a low pH, such as salami (Essid and Hassouna, 2013), whose pH values varied between 4.2 and 5.3. However, the pH values of stinky mandarin fish were higher than other fermented foods, mainly due to its shorter fermentation time. There was no significant difference between the inoculated groups and the control one, indicating that inoculation of starter culture did not have a significant effect on pH values.

**Table 1:** Changes of pH, total bacteria count, and lactic acid bacteria during the fermentation.

Samples^A^	pH			Total bacteria count (log cfu/ml)			Lactic acid bacteria (log cfu/ml)		
	1D	3D	5D	1D	3D	5D	1D	3D	5D
C	6.81 ± 0.05^a1^	6.48 ± 0.07^ab1^	6.58 ± 0.12^a1^	4.53 ± 0.01^a1^	4.82 ± 0.07^a1^	5.51 ± 0.19^a2^	4.53 ± 0.16^a2^	4.02 ± 0.04^a1^	4.61 ± 0.10^a2^
L	6.83 ± 0.04^a2^	6.50 ± 0.03^a1^	6.47 ± 0.05^a1^	6.18 ± 0.03^d1^	6.53 ± 0.03^d2^	6.62 ± 0.07^b2^	6.02 ± 0.07^c1^	6.52 ± 0.02^c2^	6.56 ± 0.11^c2^
W	6.87 ± 0.01^a3^	6.48 ± 0.01^a2^	6.57 ± 0.03^a1^	5.33 ± 0.06^b1^	5.47 ± 0.06^b1^	5.56 ± 0.08^a2^	6.14 ± 0.05^c1^	5.97 ± 0.27^b1^	5.90 ± 0.06^b1^
LW	6.97 ± 0.02^b3^	6.64 ± 0.02^b2^	6.49 ± 0.04^a1^	5.70 ± 0.02^c1^	5.78 ± 0.03^c1^	5.83 ± 0.08^a1^	5.54 ± 0.11^b1^	5.81 ± 0.08^b2^	6.00 ± 0.01^b2^

Values with unlike superscript letters (a–c) in the same column are significantly different (*p* < 0.05).

Values with unlike superscript letters (1–3) in the same row of between the fermentation process are significantly different (*p* < 0.05).

A C, L, 5, and LW represented fish fermented without starter, with *Lactoc. lactis* M10, with *W. cibaria* M3, and with both of the bacteria for 5 days.

For microorganisms, the initial amount of total bacteria in the control group was 4.53 log cfu/ml (Table 1), which gradually increased to 5.51 log cfu/ml at the end of fermentation. The initial amounts of total bacteria in L, W, and LW groups’ samples ranged from 5.33 to 6.18 log cfu/ml, which was higher than that of the control due to the inoculation of LAB. At the end of fermentation, no significant difference was observed between the total bacteria amount of the control and batches with starter, except for the L group. The same tendency was observed in LAB counting (Table 1). The number of LAB in the L group was higher than that of the other three batches, which indicated that *Lactoc. lactis* M10 could grow well in this system. The LAB amounts in W and LW groups were also higher than that of the control, which suggested the inoculated strains could adapt well to the fermentation conditions and became the dominant bacteria in the microbiota community of stinky mandarin fish. It had been shown that LAB, as the dominant bacteria, played an important role in fermented foods by utilizing carbohydrates and proteins to produce lactic acid and bacteriocins, and could improve the flavors of products and inhibit the growth of spoilage bacteria, which had a positive impact on the quality of products (Zhang et al., 2013; Gao et al., 2018). Co-inoculation of LAB greatly increased their numbers and shortened the time for LAB occupying the dominant position in *Chouguiyu*.

### Bacterial Community Succession During the Fermentation

**Figure 1:**
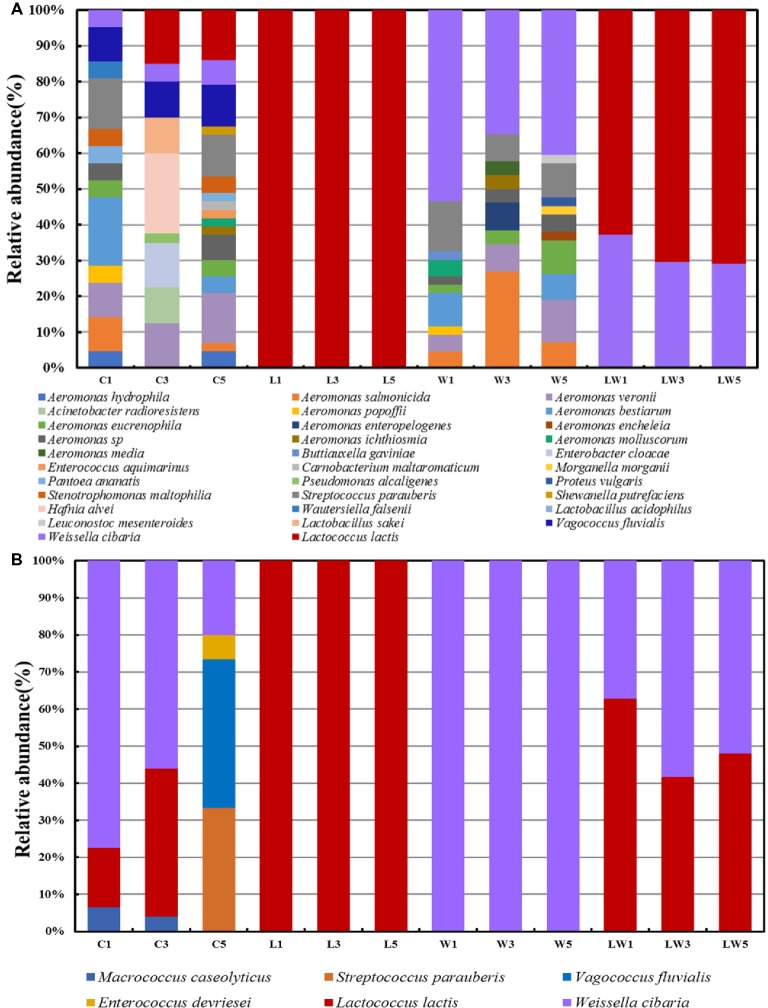
Relative bacterial composition showing the bacterial succession during the fermentation process with and without starter culture obtained on plate count agar plates **(A)** and MRS plates **(B)**.

In order to further understand the changes of bacterial succession during the fermentation process, MALDI-TOF was used to identify the bacterial compositions on plate count agar (Figure 1A) and MRS plates (Figure 1B). The bacterial diversity in the control group is more complex than that in the other three groups. This resulted from various microorganisms attached in the surface of fish body, such as *Aeromonas* (Montes et al., 1999), which were facultative anaerobic bacteria and occurred ubiquitously and autochthonously in aquatic environments. The amounts of *Lactoc. lactis* M10 and *W. cibaria* M3 increased from 4.76 to 20.93% during the fermentation on the plate count agar plate. What is more, it was observed that the increase of *Lactoc. lactis* M10 was greater than that of *W. cibaria* M3 on both plate count agar and MRS plates in the control group, implying that these two strains may have competitive relationships in the fermentation system.

For the group with starter cultures, it could be seen that the dominant bacteria were the starter culture they inoculated, *Lactoc. lactis* M10 and *W. cibaria* M3. For sample L, *Lactoc. lactis* became the only dominant bacteria on either plate count agar or MRS plates throughout the fermentation process. It was reported that *Lactoc. lactis* was capable of producing bacteriocin (Saelao et al., 2018), such as nisin, which could suppress the spoilage or pathogen bacteria. Ho et al. found that in the presence of *Lactoc. lactis* KTH0-1S, pathogenic bacteria, such as *L. monocytogenes* decreased significantly in cheese (Ho et al., 2018). For the W group, strains such as *Aeromonas* were detected in addition to *W. cibaria* M3. *A. salmonicida* (Park et al., 2017) and *Streptococcus parauberis* (Fernandez-No et al., 2012) were reported as the pathogenic bacteria in aquatic products and were commonly found in food spoilage. The reason for more spoilage bacteria found in group W may be due to the weak ability of *W. cibaria* M3 to produce bacteriocin and its limited ability to inhibit other microorganisms. It is reported that not all *W. cibaria* (Li et al., 2017) strains could produce bacteriocin, but the production capacity was quite different between strains (Woraprayote et al., 2015). In addition, *Lactoc. lactis* M10 increased with the decrease of *W. cibaria* M3 during the fermentation (Figures 1A, B), either on the plate count agar or MRS plates, indicating that there was a competitive relationship between the two strains, which was the same as that in the control group. Romi also observed in fermented bamboo shoots (Romi et al., 2015) that *W. cibaria* was gradually decreased or even disappeared with the extension of fermentation time, while *Lactoc. lactis* subsp. could be detected during the whole fermentation period. Therefore, *Lactoc. lactis* might have a competitive relationship with *W. cibaria* by occupying a dominant position in the ecosystem, and played an important role in the control of spoilage bacteria and improvement of the product quality.

### Changes of TVB-N, TBARS, and Biogenic Amines During the Fermentation

The initial TVB-N contents were in the range of 20.32–22.90 mg/100 g. Then, TVB-N values significantly increased and reached 49.49 mg/100 g at the fifth day in the control group. TVB-N concentrations in the W and LW groups were 52.68 mg/100 g and 47.49 mg/100 g, respectively, and showed no significant difference with the control group. In contrast, TVB-N content (34.17 mg/100 g) was greatly decreased in the fish inoculated with *Lactoc. lactis* M10. This result was in accordance with Hu’s (Hu et al., 2008) and Yin’s studies (Yin et al., 2002), who both reported that LAB inoculation could inhibit the accumulation of TVB-N by producing lactic acid and bacteriocins in fish or meat fermentation.

*Si. chuatsi* is a kind of fish containing highly unsaturated lipids, which are easily susceptible to oxidation and resulted in a rancid smell and taste. TBARS is often used as an indicator for the degree of lipid oxidation. It has been reported that fish is of good quality when the TBARS content is below 5 mg/kg (Zeng et al., 2013). In our study, the initial TBARS contents were 0.03–0.72 mg of MDA/kg. At the end of 5 days’ fermentation, the highest TBARS value was observed in the control group at 1.93 mg MDA/kg and sample with *Lactoc. lactis* M3 had the lowest TBARS value at 1.21 mg MDA/kg. The TBARS values of the samples with and without starter cultures differed significantly (*p* < 0.05). It was reported that LAB had antioxidant effects on unsaturated fatty acids, and suppressed increase of TBARS in fermented fish (Zeng et al., 2013) and dry sausages (Sun et al., 2017). Therefore, it was reasonable that TBARS contents for products inoculated with *Lactoc. lactis* M10 and *W. cibaria* M3 could be definitely lower than that in the control group.

Biogenic amines, such as histamine, cadaverine, putrescine and tyramine, are potentially produced due to the growth of active microorganisms, acidification, and proteolysis that generates free amino acids during the fermentation process (Gardini et al., 2016). Changes in the concentrations of biogenic amines were monitored during fish fermentation and summarized in Table 2. Histamine contents were very low and were not detected in all the samples, while significant differences were found in putrescine, tyramine, and cadaverine between the control group and starter culture groups (*p <* 0.05). Compared with the control group, putrescine reduced to 21.6, 46.9, and 39.3% for the L, W and LW group, respectively. For cadaverine, the content in the starter cultures’ were reduced to 0, 76.3 and 17.2% for each group as shown in Table 2. LAB starter culture has been commonly considered to suppress the growth of wild amine-producing bacteria, such as Enterobacteriaceae, which often contained high lysine- and ornithine-decarboxylase activities and are correlated with the production of cadaverine and putrescine (Bover-Cid et al., 2001; Durlu-Ozkaya et al., 2001). Therefore, the inoculation of *Lactoc. lactis* M3 or *W. cibaria* M3 may inhibit the non-starter LAB, such as *Enterobacter cloacae* in the control group, thus reducing the content of biogenic amines.

**Table 2:** Changes of TVB-N, TBARS, and biogenic amines during the fermentation.

Samples^A^	TVB-N (mg/100 g)			TBARS (mg/kg)			Biogenic amines (mg/kg)			
	1D	3D	5D	1D	3D	5D	Histamine	Cadaverine	Tyramine	Putrescine
C	22.67 ± 2.92^a^	47.18 ± 0.50^c^	49.49 ± 0.99^b^	0.03 ± 0.00^a^	1.38 ± 0.00^c^	1.98 ± 0.02^d^	ND^a^	3.08 ± 1.27^b^	4.85 ± 1.18^c^	25.19 ± 4.16^b^
L	22.90 ± 0.99^a^	26.23 ± 1.97^a^	34.17 ± 0.99^a^	0.10 ± 0.01^b^	1.35 ± 0.00^b^	1.21 ± 0.02^a^	ND^a^	ND^a^	ND^a^	5.46 ± 2.57^a^
W	20.32 ± 1.48^a^	35.82 ± 0.50^b^	52.68 ± 1.97^b^	0.72 ± 0.04^c^	1.12 ± 0.00^a^	1.59 ± 0.04^b^	ND^a^	2.35 ± 2.24^b^	2.72 ± 0.52^b^	11.82 ± 5.91^a^
LW	22.20 ± 1.99^a^	26.36 ± 0.00^a^	47.49 ± 2.98^b^	0.05 ± 0.00^a^	1.34 ± 0.00^b^	1.79 ± 0.10^c^	ND^a^	0.53 ± 0.52^a^	4.88 ± 0.61^c^	9.90 ± 1.99^a^

Values with unlike superscript letters (a, b) in the same column are significantly different (*p* < 0.05).

A C, L, W, and LW represent fish fermented without starter, with *Lactoc. lactis* M10, with *W. cibaria* M3, and with both of the bacteria.

### Changes in Sensor Response Signals During the Fermentation

**Figure 2:**
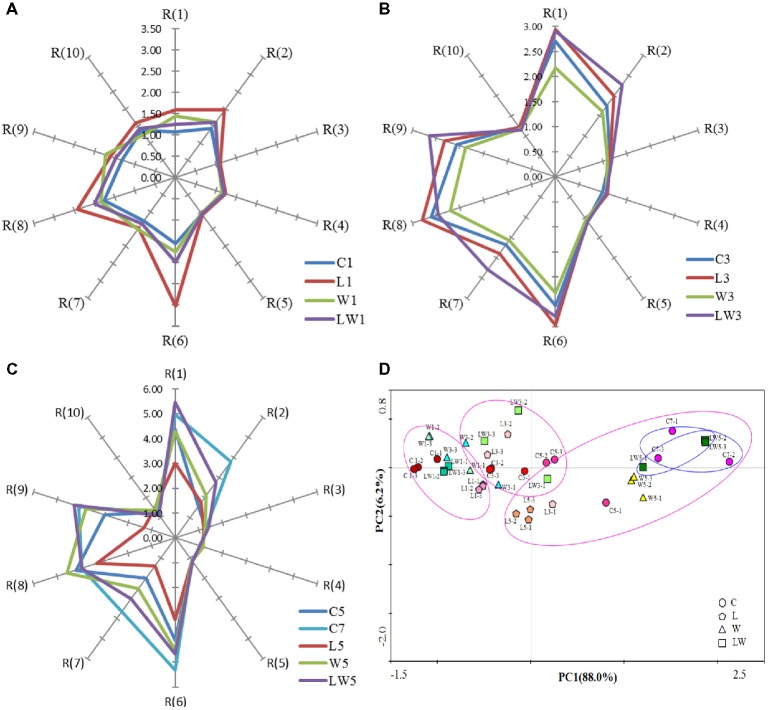
E-nose analysis and PCA plot of samples inoculated with or without starter cultures. **(A)** E-nosc evaluation on days 1. **(B)** E-nose evaluation on days 3. **(C)** E-nose evaluation on days 5 and 7. **(D)** PCA results of E-nose profile evolution during the fermentation process. C, L, W and LW represented fish fermented without starter, with *Lactoc. lactis* M10, with *W. cibaria* M3 and with both of the bacteria.

Sensor responses in *Chouguiyu* among groups were measured during fermentation and shown in Figures 2A–C at days 1, 3, and 5 respectively. Sensor response values were observed to rise with the prolongation of fermentation time, indicating that the total volatile compounds increased gradually with the fermentation process. The sensor signals of R(1), R(2), R(6), R(7), R(8), and R(9) were stronger than others, which meant that these sensors were more sensitive and the correspondent substances, which were aromatics, nitrogen oxides, and sulfur-containing organics, were increased greatly during fermentation.

The radar map of L1 samples at the early stage of fermentation (Figure 2A) was the largest with an area of 8.30, while that of control sample was minimum of 5.00. Differences among other three groups were little. At the middle stage of fermentation, the LW3 samples’ area of 11.69 was the largest in all samples. After five days’ fermentation, the sensor profile of LW5 group was the most similar to the control group fermented for seven days (C7). The area of LW5 group was the largest in all starter culture groups, accounting for 86.74% of C7. The profile of W5 group, which was the second similar to the C7 group’s, reached an area of 20.89 and accounted for 75.58% of the control group’s. In contrast, L5 had the minimum area of 9.91. This result indicated that *Lactoc. lactis* M10 played an important role at the beginning of fermentation, while *W. cibaria* M3 contributed to produce flavor substances at the later fermentation period. When *Lactoc. lactis* M10 and *W. cibaria* M3 exist at the same time, it may promote the formation of flavor substances in the LW sample, which most resembled the C7 sample, and resulted in the fermentation time shortened by 29%.

To reveal the relationship of the sample with or without starter cultures, the data of sensor response signals were processed by PCA. The first principal component explained 88.0% of the total variance, and the second principal component explained 1.54% of the total variance, indicating that the two principal components could fully reflect the characteristic information of the volatile substances in *Chouguiyu* with or without starters. At the early stage of fermentation, these four groups of samples were relatively concentrated and overlapped in their respective regions, indicating the differences between the electronic responses at the early stage of fermentation were not very significant. As the fermentation progressed, the differences were observed among the samples. At the end of fermentation, the samples of W5, LW5, and C7 were gathered separately from the other samples. Moreover, the overlap area of LW5 and C7 samples were larger than the others, indicating that the flavor of these two groups was the most similar. It could be inferred that *W. cibaria* M3 played an important role in the later stage of flavor formation, and co-inoculation of *Lactoc. lactis* M10 and *W. cibaria* M3 would promote the formation of flavor with significantly shortened time.

### Changes of Volatile Compounds Monitoring by GC-MS During the Fermentation

**Figure 3:**
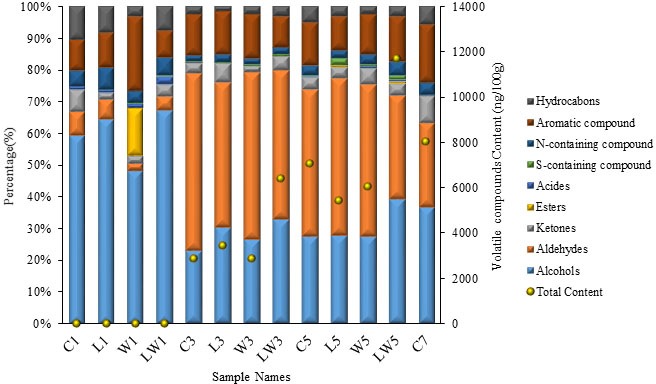
Changes of different types and contents of volatile compounds in *Chouguiyu* with or without starter cultures during the fermentation process. C, L, W and LW represented fish fermented without starter, with *Lactoc. lactis* M10, with *W. cibaria* M3 and with both of the bacteria during the fermentation process. 5 and 7 represented that fish was fermented for 5 or 7 days.

The concentrations of volatile compounds in the samples with or without starters were analyzed by GC-MS and listed in Table S1. A total of 43 volatile compounds were identified and quantified in 13 samples. Total contents of flavor substances were obviously increased (Figure 3) with the extension of fermentation time, especially for the proportion of aldehydes and aromatic substances. In the naturally fermented sample at the seventh day, volatile compounds were mainly abundant of 36.8% alcohols, 26.4% aldehydes, 18.3% aromatic compounds, and 8.5% ketones. What is more, increase of alcohols and acids was observed at the later stage of fermentation in both the C7 and LW5 groups, and volatile compound contents were significantly higher than that in the other groups. The volatile compound content in LW group at day 3 (6405.98 ng/100 g) could be comparable to that of natural fermentation at day 5 (7074.23 ng/100 g), and the content in LW group at day 5 (11702.94 ng/100 g) was even higher than that of natural fermentation at day 7 (8055.79 ng/100 g). This result indicated that the production of flavor substances was significantly enhanced by mixed inoculation, and the fermentation period was effectively shortened by 29%.

Odor activity value (OAV) was defined as dividing the concentration of the odorant in the samples by the mean values of its estimated orthonasal threshold, and was widely used to identify the major odor-active compounds in food, such as fish sauce (Lapsongphon et al., 2015), wine (de Lerma et al., 2018), and cheese (Majcher and Jelen, 2011). Depending upon the volatile compounds’ thresholds and concentrations, a total of 11 compounds including 1-octen-3-ol, α-terpineol, linalool, hexanal, heptanal, nonanal, oleic acid, indole, ethylbenzene, β-ocimene, and D-limonene, were found to have OAVs larger than 1. Consistent with our results, 1-octen-3-ol, hexanal, heptanal, nonanal, indole, and linalool were also recognized as major odor-active compounds of *Chouguiyu* in Li’s report. In addition, these compounds showed a process-dependent rising trend (Table S1), especially in the LW group. This result indicated that co-inoculation of *Lactoc. lactis* M10 and *W. cibaria* M3 had a positive effect for flavor production, especially for the aroma-active and key volatile compounds.

Aliphatic aldehydes, including hexanal, heptanal, and nonanal, were often found in fermented fish (Zeng et al., 2017), and were generally associated with unsaturated fatty acid autoxidation and enzymatic oxidation by starter microorganisms, such as *Lactob. plantarum*. These aldehydes could lead to a uniquely fatty, fishy, and nutty flavor in the *Chouguiyu* product due to their low odor thresholds. Indole has been reported to have an odor like putrid, musty, and floral on high dilutions. Liu et al. (2012) reported that indole was abundant in stinky tofu, a Chinese traditional soybean food. Similar to stinky tofu, indole was also abundant in the stinky fish product both in Li’s (2013) report and in our study. Its concentration corresponded well with time, and was significantly greater in the LW group than other three groups at the same days. This result indicated that the inoculation of *Lactoc. lactis* M10 and *W. cibaria* M3 could promote the increase of indole content in the LW group. Another aroma-active compound, linalool, probably originated from spices in the process of manufacture, also showed an increasing trend with time, particularly in the LW group with the concentration of 1937.41 ng/100 g fish meat in LW5 sample. This substance from spices may dissolve in water and gradually penetrate into the fish body and give the product a uniquely botanical and citrus flavor to improve the overall flavor quality.

**Figure 4:**
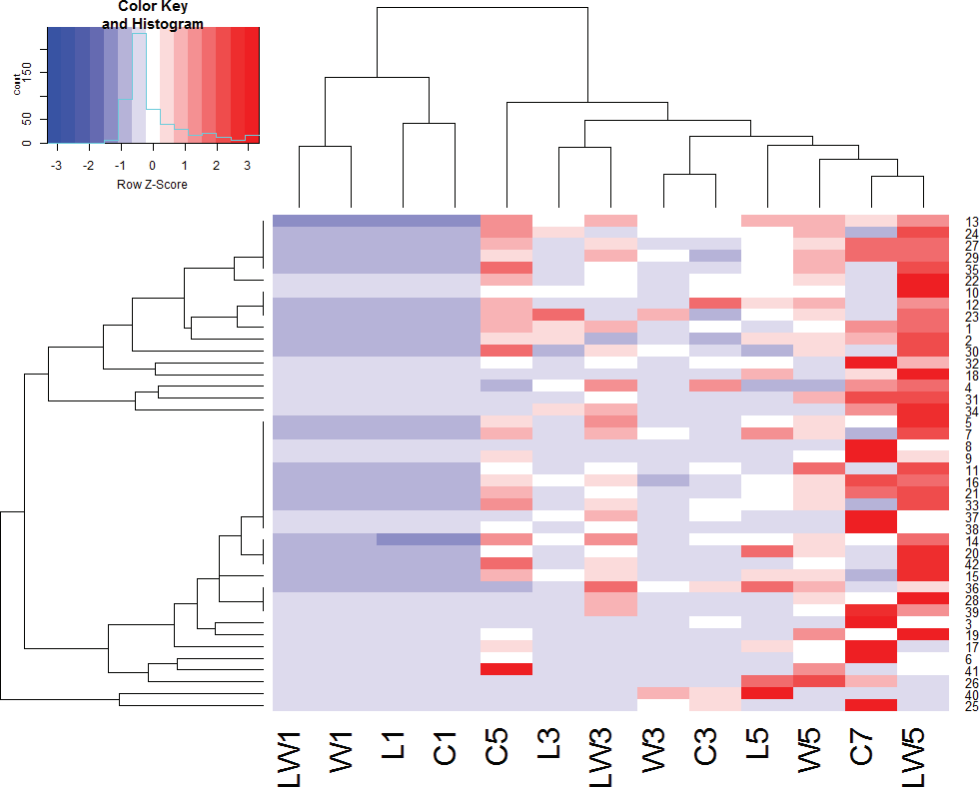
Hierarchical clustering of volatile compounds in different fermentations. C, L, W and LW represented fish fermented without starter, with *Lactoc. lactis* M10, with *W. cibaria* M3 and with both of the bacteria during the fermentation process. 1–42 represented volatile compounds in Table S1.

Hierarchical clustering analysis was performed with volatile compounds among different groups during the fermentation process. Samples appearing close in the tree are those that had a close proximity and the distance indicates the close relationship among samples. It could be deduced that flavor metabolites changed greatly at different stage. As shown in Figure 4, the volatile profiles of four samples in the initial stage of fermentation were obviously different from those in the late fermentation stages. Samples at the middle fermentation stage, which were C3, L3, W3, and LW3, clustered relatively close. Samples in the late fermentation stage were closer to naturally fermented product for 7 days. LW5 and C7 were classified in one cluster and showed similar metabolic activities. They were both characterized by high production of 1-octen-3-ol, α-terpineol, 1-octanol, γ-terpineol, 2-methyl-3-octanone, indole, 4-ethyl-o-xylene, anethole, pentamethyl, (1-methoxy-4-methyl-3-pentenyl)-benzene, and D-Limonene. The hierarchical clustering result was similar to that of PCA analysis based on E-nose measurement, and indicated that LW5 and C7 were the most similar. Therefore, GC-MS results showed that inoculation of *Lactoc. lactis* M10 and *W. cibaria* M3 could promote the flavor formation in *Chouguiyu* products with time shortened.

### Sensory Evaluation

In order to evaluate the acceptability of the product with or without starter cultures, sensory analysis, including appearance, aroma, taste, tactile texture, and overall acceptance, was carried out among the four groups (Table 3). There were no significant differences (*p* < 0.05) in the appearance and the texture of the products among four groups, indicating that inoculation of starter cultures did not affect appearance and texture. The activity of endogenous enzyme is the main factor contributing to the product’s texture, such as the development of tenderness in beef (Cruzen et al., 2014). Therefore, it was easy to understand that microbial inoculation did not affect the texture of the product. For aroma and taste evaluation, the L group had the lowest score, and was significantly different (*p* < 0.05) compared with the control group. The scores of LW group, both in aroma and taste, were the closest ones to C7 (*p* < 0.05). Zeng et al. (2017) reported that the quality of flavor compounds was remarkably improved by adding mixed starter cultures to *Suanyu* compared with that without inoculation. Xu et al. (2018) also reported that the formation of volatile compounds, which are essential for the distinct aroma in fermented fish, was mainly from microorganisms rather than endogenous enzymes. The overall acceptability in the LW group was higher than that in L or W group, and was the most similar to the naturally fermented group. This result indicated that co-inoculation of *Lactoc. lactis* M10 and *W. cibaria* M3 was able to promote the generation of flavor, improve the acceptability, and shorten the fermentation period at the same time.

**Table 3:** Sensory evaluation (mean panel score) of *Chouguiyu* by different starter cultures and control.

Attribute	C7	L5	W5	LW5
Appearance	7.15 ± 0.42^a^	7.15 ± 0.79^a^	7.21 ± 0.62^a^	7.21 ± 0.63^a^
Aroma	2.39 ± 0.68^b^	1.57 ± 0.45^a^	1.92 ± 0.72^ab^	2.39 ± 0.68^b^
Taste	6.42 ± 0.64^b^	5.28 ± 0.80^a^	5.67 ± 0.50^a^	6.54 ± 0.69^b^
Tactile texture	4.86 ± 0.99^a^	4.73 ± 0.98^a^	4.63 ± 0.93^a^	4.74 ± 0.88^a^
Overall acceptance	7.40 ± 0.42^b^	6.64 ± 0.70^a^	6.83 ± 0.39^a^	7.59 ± 0.41^b^

Values with different letters (a, b) in the same row are significantly different (*p* < 0.05).

## Conclusions

Two indigenous LAB, *Lactoc. lactis* M10 and *W. cibaria* M3, were used to enhance the flavor production and quality properties of *Chouguiyu* product. Results showed that inoculation could accelerate the LAB dominant process and there might be some competitive relationship between *Lactoc. lactis* M10 and *W. cibaria* M3. LAB inoculation, especially in *Lactoc. lactis* M10, contributed to suppress the TVB-N, TBARS, and formation of biogenic amines in the final product. Flavor formation was firstly depended on *Lactoc. lactis* M10 and later on *W. cibaria* M3. Co-inoculation of these two could promote the flavor production and resulted in LW5 most resembling C7 in both E-nose and GC-MS analyses. Therefore, co-inoculation of *Lactoc. lactis* M10 and *W. cibaria* M3 in *Chouguiyu* could enhance the flavor formation, quality attributes as well as overall acceptance and shorten fermentation time.

## Author Contributions

XL, BZ, SY, and XY conceived and planned the experiments. RB, SL, CJ, and HL performed the experiments. XL, BZ, and RB took the lead in writing the manuscript. All the above authors contributed to the interpretation of the results, provided critical feedback and helped shape the research, analysis and manuscript. YZ and XL revised the manuscript and proofed the final version of publication.

### Conflict of Interest Statement

The authors declare that the research was conducted in the absence of any commercial or financial relationships that could be construed as a potential conflict of interest.
