# Supplementary material for: Shortening Fermentation Period and Quality Improvement of Fermented Fish, Chouguiyu, by Co-inoculation of Lactococcus lactis M10 and Weissella cibaria M3
Source: Front Microbiol. 2018 Dec 17;9:3003. doi: 10.3389/fmicb.2018.03003 (PMC6327836; doi:10.3389/fmicb.2018.03003)
Supplement: Supplementary file 1 [file Table_1.DOCX]

**Table S1** Volatile compounds of *Chouguiyu* samples inoculated with or without starter culture during the fermentation process.

| ID | Volatile compounds | Chemical formula | Threshold (ng/100 g)^B^ | Content (ng/100g) | | | | | | | | | | | | |
| --- | --- | --- | --- | --- | --- | --- | --- | --- | --- | --- | --- | --- | --- | --- | --- | --- |
|  |  |  |  | C1 | L1 | W1 | LW1 | C3 | L3 | W3 | LW3 | C5 | L5 | W5 | LW5 | C7 |
| 1 | 1-Octen-3-ol | C_8_H_16_O | 0.67 | 0 | 0 | 0.03 | 0.03 | 154.65 | 231.83 | 100.03 | 347.33 | 302.29 | 114.16 | 209.84 | 483.61 | 379.71 |
| 2 | α-terpineol | C_10_H_18_O | 6.67 | 0 | 0.07 | 0.06 | 0 | 46.67 | 340.81 | 126.4 | 0 | 330.37 | 330.54 | 284.71 | 745.45 | 389.36 |
| 3 | 5-Octen-1-ol | C_8_H_16_O |  | 0.16 | 0.11 | 0.05 | 0.04 | 46.29 | 0 | 1.43 | 0 | 14.55 | 2.65 | 2.85 | 30.18 | 562.74 |
| 4 | 1-Octanol | C_8_H_18_O | 3.33 | 3.95 | 4.24 | 3.09 | 3 | 62.05 | 16.46 | 4.43 | 57.05 | 0 | 0 | 0 | 70.87 | 63.78 |
| 5 | linalool | C_10_H_18_O | 0.27 | 1.49 | 1.12 | 0.18 | 0.09 | 143.35 | 237.88 | 284.83 | 1124.71 | 699.92 | 436.47 | 656.36 | 1937.41 | 434.23 |
| 6 | Bicyclo[4.1.0]heptan-3-ol, 4,7,7-trimethyl- | C_10_H_18_O |  | 0.05 | 0.09 | 0.02 | 0.02 | 0 | 1.6 | 0 | 0 | 11.37 | 0 | 10.41 | 17.8 | 271.83 |
| 7 | Bicyclo[3.1.1]hept-3-en-2-ol, 4,6,6-trimethyl-, [1S-(1α,2β,5α)]- | C_10_H_16_O | 2000 | 1.87 | 1.59 | 1.13 | 1.02 | 144.12 | 152.46 | 202.28 | 482.22 | 464.68 | 534.01 | 369.11 | 801.32 | 12.17 |
| 8 | terpinen-4-ol | C_10_H_18_O | 2000 | 1.92 | 1.21 | 0.2 | 0.12 | 1.74 | 1.75 | 1.61 | 4.82 | 6.37 | 5.21 | 7.65 | 44.76 | 674.06 |
| 9 | γ-terpineol | C_10_H_18_O |  | 0.91 | 0.36 | 0.11 | 0.11 | 5.4 | 2.92 | 3.18 | 8.75 | 37.52 | 6.92 | 15.09 | 51.08 | 166.17 |
| 10 | p-Cymen-7-ol | C_10_H_14_O |  | 0 | 0 | 1.91 | 0 | 52.91 | 50.06 | 33.44 | 64.65 | 82.29 | 67.89 | 68.16 | 365.14 | 7 |
| 11 | D-arabino-Hex-1-enitol, 1,5-anhydro-2-deoxy- | C_6_H_10_O_4_ |  | 1.57 | 0.74 | 0.49 | 0.54 | 12.62 | 5.69 | 6.29 | 14.14 | 13.16 | 7.91 | 44.08 | 52.63 | 7.79 |
|  | ***Alcohols*** | **∑** |  | **11.92** | **9.53** | **7.27** | **4.97** | **669.8** | **1041.46** | **763.92** | **2103.67** | **1962.52** | **1505.76** | **1668.26** | **4600.25** | **2968.84** |
| 12 | Benzaldehyde | C_7_H_6_O | 220 | 0 | 0 | 0.01 | 0 | 106.96 | 15.68 | 21.93 | 22.66 | 74.54 | 50.11 | 70.33 | 87.62 | 26.25 |
| 13 | Hexanal | C_6_H_12_O | 0.55 | 0 | 0 | 0 | 0 | 1221.47 | 1213.79 | 1294.08 | 2338.22 | 2514.09 | 2179.49 | 2282.8 | 2505.26 | 1950.56 |
| 14 | Heptanal | C_7_H_14_O | 6.00 | 0 | 0.01 | 0.09 | 0.13 | 114.57 | 129.33 | 64.87 | 272.13 | 267.45 | 107.29 | 195.37 | 318.44 | 147.16 |
| 15 | Nonanal | C_9_H_18_O | 0.63 | 1.51 | 0.93 | 0.24 | 0.19 | 151.13 | 205.03 | 115.05 | 377.79 | 404.78 | 343.8 | 351.95 | 883.83 | 0 |
|  | ***Aldehydes*** | **∑** |  | **1.51** | **0.94** | **0.34** | **0.32** | **1594.13** | **1563.83** | **1495.93** | **3010.8** | **3260.86** | **2680.69** | **2900.45** | **3795.15** | **2123.97** |
| 16 | 2-methyl-3-Octanone | C_9_H_18_O |  | 1.37 | 0.33 | 0.23 | 0.2 | 82.3 | 193.54 | 48.05 | 265.17 | 226.12 | 155.63 | 280.38 | 456.64 | 551.73 |
| 17 | piperitone | C_11_H_18_O |  | 0.08 | 0 | 0.18 | 0.09 | 6.9 | 7.16 | 6.1 | 0 | 31.22 | 27.48 | 26.35 | 0 | 134.44 |
|  | ***Ketones*** | **∑** |  | **1.45** | **0.33** | **0.41** | **0.29** | **89.2** | **200.7** | **54.15** | **265.17** | **257.34** | **183.11** | **306.73** | **456.64** | **686.17** |
| ID | Volatile compounds | Chemical formula |  | Content (ng/100g) | | | | | | | | | | | | |
|  |  |  |  | C1 | L1 | W1 | LW1 | C3 | L3 | W3 | LW3 | C5 | L5 | W5 | LW5 | C7 |
| 18 | 2-Propenoic acid, butyl ester | C_7_H_12_O_2_ |  | 0 | 0 | 2.23 | 0 | 4.32 | 0 | 0 | 4.67 | 0 | 26.34 | 0 | 71.02 | 17.42 |
|  | ***Esters*** | **∑** |  | **0** | **0** | **2.23** | **0** | **4.32** | **0** | **0** | **4.67** | **0** | **26.34** | **0** | **71.02** | **17.42** |
| 19 | Oleic Acid | C_18_H_34_O_2_ | 0.67 | 0.16 | 0.15 | 0.16 | 0.18 | 2.18 | 1.61 | 0 | 0 | 12.03 | 3.5 | 32.98 | 65.79 | 9.24 |
|  | ***Acides*** | **∑** |  | **0.16** | **0.15** | **0.16** | **0.18** | **2.18** | **1.61** | **0** | **0** | **12.03** | **3.5** | **32.98** | **65.79** | **9.24** |
| 20 | Methyl thiolacetate | C_3_H_6_OS |  | 0 | 0.02 | 0.05 | 0.03 | 13.35 | 8.16 | 17.77 | 42.97 | 25.46 | 116.22 | 46.39 | 161.94 | 4.38 |
|  | ***S-containing compound*** | **∑** |  | **0** | **0.02** | **0.05** | **0.03** | **13.35** | **8.16** | **17.77** | **42.97** | **25.46** | **116.22** | **46.39** | **161.94** | **4.38** |
| 21 | Indole | C_9_H_7_NO | 0.02 | 1.02 | 1 | 0.6 | 0.42 | 39.03 | 75.5 | 49.73 | 133.37 | 194.91 | 136.92 | 148.23 | 366.09 | 294.08 |
| 22 | Benzene, 1-methyl-2-(1-methyl-2-propenyl)- | C_8_H_15_N_3_ |  | 0 | 0.04 | 0 | 0 | 14.77 | 6.78 | 6.1 | 19.1 | 46.89 | 16.09 | 35.05 | 135.87 | 3.19 |
|  | ***N-containing compound*** | **∑** |  | **1.02** | **1.04** | **0.6** | **0.42** | **53.8** | **82.28** | **55.83** | **152.47** | **241.8** | **153.01** | **183.28** | **501.96** | **297.27** |
| 23 | o-Xylene | C_8_H_10_ | 666.67 | 0 | 0 | 2.67 | 0 | 23.57 | 236.92 | 161.51 | 47.95 | 159.93 | 82.81 | 124.59 | 227.85 | 38.35 |
| 24 | Ethylbenzene | C_8_H_10_ | 6.67 | 0 | 0 | 0 | 0 | 55.29 | 88.44 | 51.28 | 29.3 | 126.75 | 46 | 98.05 | 188.23 | 7.18 |
| 25 | 3,5-Dimethylanisole | C_9_H_12_O |  | 0.11 | 0.04 | 0 | 0 | 10.51 | 0 | 4.68 | 0 | 0 | 0 | 0 | 0 | 62.5 |
| 26 | [β-Cymene](javascript:showMsgDetail('ProductSynonyms.aspx?CBNumber=CB9461741&postData3=CN&SYMBOL_Type=A');) | C_10_H_14_ |  | 0.03 | 0.05 | 0.01 | 0.01 | 0 | 0 | 0 | 0 | 0 | 70.53 | 82.64 | 0 | 45.98 |
| 27 | 4-ethyl-[o-Xylene](javascript:showMsgDetail('ProductSynonyms.aspx?CBNumber=CB9415092&postData3=CN&SYMBOL_Type=A');) | C_10_H_14_ |  | 0 | 0 | 0 | 0 | 30.23 | 24.99 | 38.31 | 98.39 | 137.11 | 74.76 | 104.55 | 212.53 | 191.7 |
| 28 | 2H-1-Benzopyran, 3,4-dihydro- | C_9_H_10_O |  | 0.6 | 0.57 | 0.24 | 0.23 | 6.08 | 5.34 | 5.64 | 23.77 | 0 | 5.42 | 17.05 | 54.17 | 11.03 |
| 29 | Anethole | C_10_H_12_O |  | 0 | 0 | 0 | 0 | 10.12 | 12.32 | 32.79 | 65.46 | 48.79 | 33.06 | 68.12 | 114.78 | 109.41 |
| 30 | 5-Isopropyl-m-xylene | C_11_H_16_ |  | 0 | 0 | 0 | 0 | 11.86 | 0 | 16.61 | 42.25 | 76.61 | 0 | 38.12 | 90.38 | 5.46 |
| 31 | 4-tert-Butyltoluene | C_11_H_16_ |  | 0.28 | 0.05 | 0 | 0.01 | 0 | 0 | 4.28 | 11.82 | 0 | 0 | 33.09 | 65.88 | 67.48 |
| 32 | Pentamethy | C_11_H_16_ |  | 0 | 0 | 0 | 0 | 122.19 | 55.21 | 0 | 124.23 | 151.86 | 92.21 | 0 | 323.2 | 912.93 |
| 33 | Naphthalene, 2-methyl- | C_11_H_10_ |  | 0.47 | 0.7 | 0.42 | 0.25 | 53.37 | 18.81 | 35.62 | 79.63 | 120.55 | 55.39 | 81.7 | 187.57 | 4.34 |
| 34 | (1-methoxy-4-methyl-3-pentenyl)-benzene | C_13_H_18_O |  | 0.1 | 0.03 | 0 | 0 | 0 | 2.74 | 0 | 2.99 | 0 | 0 | 0 | 7.08 | 3.86 |
| ID | Volatile compounds | Chemical formula |  | Content (ng/100g) | | | | | | | | | | | | |
|  |  |  |  | C1 | L1 | W1 | LW1 | C3 | L3 | W3 | LW3 | C5 | L5 | W5 | LW5 | C7 |
| 35 | Naphthalene, 1,7-dimethyl- | C_12_H_12_ |  | 0 | 0 | 0 | 0 | 15.04 | 15.29 | 19.12 | 29.35 | 122.78 | 48.11 | 76.75 | 154.03 | 7.13 |
| 36 | Naphthalene, 2,7-dimethyl- | C_12_H_12_ |  | 0.38 | 0.18 | 0.15 | 0.12 | 32.66 | 13.94 | 22.96 | 65.13 | 0 | 59.06 | 42.55 | 26.87 | 7.67 |
|  | ***Aromatic compound*** | **∑** |  | **1.97** | **1.62** | **3.49** | **0.62** | **370.92** | **474** | **392.8** | **620.27** | **944.38** | **567.35** | **767.21** | **1652.57** | **1475.02** |
| 37 | β-Ocimene | C_10_H_16_ | 6.67 | 0.09 | 0.11 | 0.06 | 0.03 | 23.27 | 16.5 | 6.8 | 46.96 | 11.46 | 10.43 | 10.41 | 29.03 | 132.97 |
| 38 | 1,3,8-p-Menthatriene | C_10_H_14_ |  | 0.65 | 0.32 | 0.06 | 0.11 | 4.69 | 3.89 | 2.64 | 12.02 | 29.56 | 8.72 | 9.99 | 22.99 | 170.55 |
| 39 | D-Limonene | C_10_H_16_ | 6.67 | 1.29 | 0.63 | 0.26 | 0.31 | 3.54 | 13.56 | 10.04 | 63.54 | 0 | 17.76 | 25.9 | 79.21 | 138.02 |
| 40 | 1-Cyclohexyl-1-pentyne | C_11_H_18_ |  | 0.1 | 0 | 0 | 0 | 23.62 | 0 | 37.78 | 0 | 0 | 107.7 | 0 | 0 | 0 |
| 41 | Di-epi-.alpha.-cedrene | C_15_H_24_ |  | 0 | 0.12 | 0.02 | 0.02 | 0 | 0 | 0 | 0 | 146.54 | 3.53 | 73.56 | 9.96 | 8.12 |
| 42 | Heptadecane | C_17_H_36_ |  | 0 | 0.06 | 0.11 | 0.1 | 20.93 | 19.25 | 22 | 83.44 | 182.28 | 34.5 | 46 | 256.43 | 23.82 |
|  | ***Hydrocabons*** | **∑** |  | **2.13** | **1.24** | **0.51** | **0.57** | **76.05** | **53.2** | **79.26** | **205.96** | **369.84** | **182.64** | **165.86** | **397.62** | **473.48** |
|  | ***Total*** |  |  | **20.16** | **14.87** | **15.06** | **7.4** | **2873.75** | **3425.24** | **2859.66** | **6405.98** | **7074.23** | **5418.62** | **6071.16** | **11702.94** | **8055.79** |
|  | ***Numbers of Volatile compounds*** |  |  | **24** | **28** | **30** | **26** | **37** | **34** | **35** | **33** | **32** | **36** | **36** | **38** | **40** |

^A^ C, L, W and LW represented fish fermented without starter, with *L.lactis* M10, with *W.cibaria* M3 and with both of the bacteria for different days.

**Table S2** The sensors array of electronic nose PEN 3

| Number in array | sensor | Performance characteristics |
| --- | --- | --- |
| R(1) | W1C | Aromatic–aliphatic compounds |
| R(2) | W5S | Oxynitride |
| R(3) | W3C | Ammonia, Aroma compounds |
| R(4) | W6S | Hydrogen |
| R(5) | W5C | Hydrocarbons, Aroma compounds |
| R(6) | W1S | Methane |
| R(7) | W1W | Sulfide |
| R(8) | W2S | Alcohol |
| R(9) | W2W | Organic sulfur compounds, Aroma compounds |
| R(10) | W3S | Hydrocarbons |
